# Molecular determination of epidermal growth factor receptor in normal and neoplastic colorectal mucosa

**DOI:** 10.1038/sj.bjc.6603441

**Published:** 2006-10-31

**Authors:** G Piazzi, P Paterini, C Ceccarelli, M A Pantaleo, G Biasco

**Affiliations:** 1 Centre of Applied Biomedical Research (CRBA), S Orsola-Malpighi Hospital, University of Bologna, Via Massarenti 9, Bologna 40138, Italy; 2 Surgical Pathology Unit, Department of Internal Medicine and Gastroenterology, S Orsola-Malpighi Hospital, University of Bologna, Via Massarenti 9, Bologna 40138, Italy; 3 Department of Oncology, Institute of Haematology and Medical Oncology, S Orsola-Malpighi Hospital, University of Bologna, Via Massarenti 9, Bologna 40138, Italy

**Keywords:** EGFr, phosphorylated EGFr, colorectal cancer, targeted therapy

## Abstract

The epidermal growth factor receptor (EGFr) is considered a major target for treatment of colorectal cancer (CRC). We found a mean EGFr content significantly lower but more activated in colonic neoplastic tissue than in paired normal mucosa. Phosphorylated (pY1068) EGFr detection in CRC may be a better tool than EGFr detection to select patients for targeted therapies.

Epidermal growth factor receptor (EGFr) plays a pivotal role in regulating epithelial proliferation, differentiation and survival. Aberrant regulation of EGFr signalling has been implicated in the development and progression of several different solid tumours (Rubin Grandis *et al*, 1996; Rusch *et al*, 1997; Goldstein and Armin, 2001). Colorectal cancer (CRC) is considered a tumour showing EGFr overexpression, but EGFr expression ranges between 25 and 75% (Salomon *et al*, 1995; Goldstein and Armin, 2001). The discrepancy in this range could be due to several factors. The first is related to EGFr detection. The most popular method of EGFr determination is immunohistochemistry (IHC) that *per se* does not provide for reliable quantitative analysis, and standardised techniques and scoring systems are lacking (Dei Tos and Ellis, 2005). The second factor is related to possible nonuniform EGFr expression within the tumour area (Goldstein and Armin, 2001). In addition, most studies are confined to neoplastic tissue without normalisation to the corresponding normal mucosa. Nevertheless, some IHC studies measuring EGFr expression both in neoplastic tissue and paired normal colonic mucosa reported a similar EGFr content in the two compartments (Moorghen *et al*, 1990; Maurer *et al*, 1998). These factors could be responsible for the lack of comparable results in literature and the lack of correlation between EGFr expression and the clinical activity of EGFr inhibitors in humans (Cunningham *et al*, 2004; Chung *et al*, 2005).

The aim of the present study was to shed more light on EGFr expression in CRC, evaluating total and activated (pY1068) EGFr and mRNA content on neoplastic tissue and paired normal mucosa using two quantitative techniques (ELISA and real-time PCR).

## MATERIALS AND METHODS

### Patients and tissues

Thirty-nine patients (21 men and 18 women) surgically treated for sporadic CRC were enrolled in the study. The clinical and pathological parameters are summarised in Table 1. Tissue samples were collected from the primary tumour and from mucosa with a roughly normal appearance more than 25 cm from the lesion. Neoplastic tissue was quickly frozen in liquid nitrogen and stored at −80°C until use. Normal mucosa was split into two blocks, one frozen and stored immediately and the other processed for histological diagnosis to exclude pathological lesions.

### Protein extraction and ELISA quantification of total and activated (pY1068) EGFr

Frozen tissues were homogenised using lysis buffer (50 mM Tris pH 7.4, 150 mM NaCl, 2 mM MgCl_2_, 1% Triton X-100, 10% glycerol, 2 mM EGTA, 1 mM DTT) containing protease inhibitors (10 mg ml^−1^ aprotinin and leupeptin, 5 mg ml^−1^ pepstatin, 1 mM PMSF) and phosphatase inhibitors (50 mM NaF, 10 mM Na_4_P_2_O_7_, 1 mM Na_3_VO_4_, 3 mM H_2_O_2_). Homogenates were centrifuged at 13 000 **g** for 15 min at 4°C, and supernatants were stored at −80°C until analysis.

The concentration of total and pY1068 EGFr was assessed using ELISA kits purchased from Biosource International Inc. (Camarillo, CA, USA). Protein lysates from A431 and SW620 cell lines were used as positive and negative controls, respectively, to verify the specificity of the EGFr ELISA assays.

Relative activated (pY1068) EGFr was defined as pY1068/total EGFr.

### RNA extraction and real-time PCR quantification of EGFr

Total RNA was isolated using TRIzol Reagent (Invitrogen Life Technologies, Carlsbad, CA, USA) and reverse transcribed using Superscript II (Invitrogen Life Technologies) with oligo-dT primers, according to the manufacturer's guidelines.

Gene-specific primers and TaqMan probes (Table 2) were designed with the Beacon Designer 2.00 Software (Premier Biosoft International, Palo Alto, CA, USA) and real-time PCR was performed using an iCycler apparatus (Bio-Rad Laboratories, Hercules, CA, USA). The cycle numbers were recorded when the accumulated PCR products crossed an arbitrary threshold (*C*_T_ or threshold cycle) and *C*_T_ values were used to calculate the expression levels of EGFr relative to the internal reference *β*-actin.

### Statistical analysis

Statistical calculations were performed using StatView 5.0 statistical software (SAS Institute Inc., Cary NC, USA). For all analyses, a *P*-value of less than 0.05 was considered significant. Values were given as mean±s.e.

The paired *t*-test was used to detect differences in total and relative activated (pY1068) EGFr and mRNA mean values between normal and neoplastic tissue. Associations between these variables were assessed by the Spearman rank test and regression analysis was performed. The relationship with clinicopathologic parameters was explored using the unpaired *t*-test.

## RESULTS

### Evaluation of total and activated (pY1068) EGFr by ELISA

**Figure 1:**
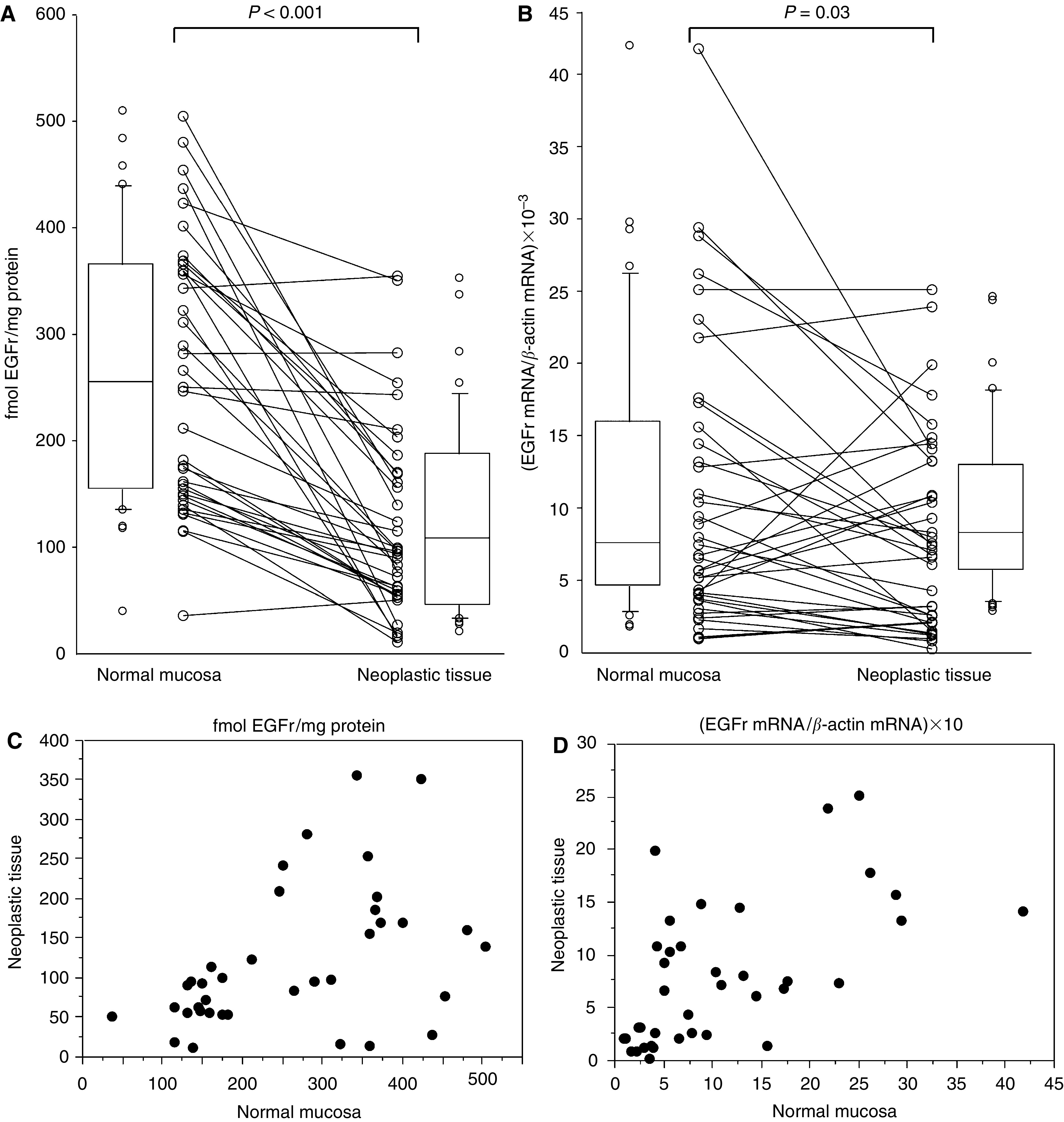
EGFr content in normal mucosa and in neoplastic tissue. Paired protein (**A**) and mRNA (**B**) samples are connected by lines. Box-plot graphic analyses are also shown. Scatterplot graphic analysis for EGFr protein (**C**) and mRNA (**D**) content are reported. Each dot represents EGFr protein or mRNA content in normal mucosa and neoplastic tissue of the same patient.

Total and activated (pY1068) EGFr levels were determined in 39 paired samples. Mean total EGFr protein content was significantly higher in normal mucosa than in neoplastic tissue ((260.8±20.3) *vs* (120.5±14.7) fmol EGFr mg^−1^ of total protein) (Figure 1A). According to Spearman's rank test, total EGFr expression levels showed an association between normal mucosa and the respective neoplastic tissue (*R*=0.49; *P*=0.004). This relationship was visualised using scatterplot graphic analysis (Figure 1C). Mean relative activated (pY1068) EGFr content was significantly higher in neoplastic tissue than in normal mucosa ((1.103±0.387) *vs* (0.129±0.019) U fmol^−1^ EGFr).

### Evaluation of EGFr mRNA expression by real-time PCR

EGFr mRNA content was determined in 39 paired samples. Mean EGFr mRNA content was significantly higher in normal mucosa than in neoplastic tissue ((10.7±1.6) × 10^−3^ *vs* (7.8±1.1) × 10^−3^) (Figure 1B). According to Spearman's rank test, EGFr mRNA expression showed an association between normal mucosa and the respective neoplastic tissue (*R*=0.65; *P*<0.0001). This relationship was visualised using scatterplot graphic analysis (Figure 1D). Regression analysis was also performed (*P*<0.0001).

### Correlation with clinicopathologic parameters

No association was found between total or relative activated (pY1068) EGFr or mRNA content in neoplastic tissue and normal mucosa and sex, tumour location, histological grading and stage.

## DISCUSSION

We studied total and activated (pY1068) EGFr and mRNA content in neoplastic tissue and paired normal mucosa samples from 39 CRC patients using two quantitative techniques. Three main results emerged from the study. Firstly, both EGFr protein and mRNA content varied widely in both neoplastic tissue and normal mucosa in our CRC patients, in agreement with IHC studies (Salomon *et al*, 1995; Goldstein and Armin, 2001). Secondly, EGFr expression in the apparently normal colorectal mucosa of our CRC patients supports the idea that EGFr expression may not be exclusive to CRC. In fact, normalising EGFr protein content in neoplastic tissue *vs* normal mucosa did not disclose EGFr overexpression. In addition, an association was found for EGFr protein or mRNA content in neoplastic tissue and normal mucosa from the same patient. Thirdly, in neoplastic tissue EGFr is more activated than in paired normal mucosa.

These results have certain biological and clinical implications. The fact that the mean EGFr content is higher in normal mucosa than in neoplastic tissue, both for protein and mRNA determination, does not exclude a role for EGFr in the carcinogenesis of CRC. Epidermal growth factor receptor signalling in the maintenance of carcinomas during intestinal tumorigenesis has been found in animal experiments (Roberts *et al*, 2002). In our study, the inverse relationship between EGFr expression and activity, demonstrated so far in CRC cell lines (Keese *et al*, 2005), supports the hypothesis that activated EGFr has a role in CRC.

In conclusion, CRC does not invariably overexpress EGFr and, most importantly, the EGFr content is frequently lower but more activated in cancer tissue than in paired normal mucosa. Epidermal growth factor receptor activity may be considered a better indicator than EGFr content when selecting expected responders to targeted therapy. This would, in part, explain the modest results observed in clinical practice when specific antibodies (i.e. Cetuximab) are combined with chemotherapy for CRC patients with IHC-positive tumors (Cunningham *et al*, 2004; Chung *et al*, 2005). The activity of specific monoclonal antibodies on apparently normal colorectal mucosa overexpressing EGFr, and the crosstalking of different biological pathways leading to cancer growth should be topics for preclinical research. Some studies currently in progress on these issues are expected to yield more data for a more correct clinical use of the so-called targeted therapies (Pantaleo *et al*, 2006).

## Figures and Tables

**Table 1:** Demographic and pathological characteristics of the CRC patients

	**Patients**	
**Characteristic**	**No.**	**%**
*Age (years)*		
Mean	69.2±1.5	
Range	46–84	
		
*Sex*		
Female	18	46.2
Male	21	53.8
		
*Lesion site*		
Right colon	15	38.5
Left colon	24	61.5
		
*Tumour diameter (mm)*		
Mean	47.4±2.5	
Range	24–100	
		
*Histological grading*		
Poorly differentiated	6	15.4
Moderately differentiated	31	79.5
Well differentiated	2	5.1
		
*Pathological staging*		
Stage II	18	46.2
Stage III	19	48.7
Stage IV	2	5.1

CRC=colorectal cancer.

**Table 2:** Primers and Taqman probes

**Gene**	**GenBank Accession No.**	**Forward primer (5′–3′)**	**Reverse primer (5′–3′)**	**Taqman probe (5′–3′)**
EGFr	NM_005228	TTCCTCCCAGTGCCTGAAT	GGGCTGGACAGTGTTGAGAT	CGCCCAGCAGAGACCCACACTACC
*β*-Actin	NM_001101	CGGCCAGGTCATCACCATTG	TGGAGTTGAAGGTAGTTTCGTGG	TGCCACAGGACTCCATGCCCAGG

EGFr=epidermal growth factor receptor.
